# A case of Sandhoff disease caused by a novel β-hexosaminidase B (HEXB) mutation c.118delG (p.A40fs*24): A case report from China

**DOI:** 10.1097/MD.0000000000033890

**Published:** 2023-06-16

**Authors:** Hongyan Xie, Shuangzhu Lin, Yang Chen, Wanqi Wang, Yangfan Qi, Jiayi Li, Qiandui Chen, Xiaochun Feng

**Affiliations:** a Diagnosis and Treatment Center for Children, Affiliated Hospital of Changchun University of Chinese Medicine, Changchun, China; b Changchun University of Chinese Medicine, Changchun, China.

**Keywords:** epilepsy, frameshift mutation, global developmental delay, *HEXB*, Sandhoff disease

## Abstract

Sandhoff disease (SD, Online Mendelian Inheritance in Man: 268800) is an autosomal recessive lysosomal storage disorder caused by variants of the β-hexosaminidase B (HEXB) gene (Online Mendelian Inheritance in Man: 606873). The HEXB gene has been mapped to chromosome 5q13 and contains 14 exons. The symptoms of SD include progressive weakness, intellectual disability, visual and hearing impairment, exaggerated startle response, and seizures; the patients usually die before the age of 3 years.^[1]^

**Case summary:**

We present a case of SD caused by a homozygous frameshift mutation in the HEXB gene, c.118delG (p.A40fs*24). The male child, aged 2 years 7 months, showed movement retrogression with orbital hypertelorism at age 2 years, accompanied by seizures. Magnetic resonance imaging of the head showed cerebral atrophy and delayed myelination of the white matter of the brain.

**Conclusion:**

A novel homozygous frameshift c.118delG (p.A40fs*24) variant of HEXB has caused SD in the child. The major symptoms are intellectual disability, visual and hearing impairment, and seizures. Investigation will be continued in the future to comprehensively describe the genotype/phenotype and gain information on other associated features to understand the variable expressivity of this condition.

## 1. Introduction

Sandhoff disease (SD; Online Mendelian Inheritance in Man: 268800) is an autosomal recessive disorder. SD is one of a group of autosomal recessive disorders known as GM2 gangliosidosis disease. Generally, GM2 gangliosides are catabolized by β-hexosaminidase A (HEXA). This enzyme consists of 2 subunits, which are encoded by HEXA and β-hexosaminidase B (HEXB) genes. Mutations in either of these genes cause the accumulation of GM2 gangliosides. If both parents carry pathogenic variants of the HEXB gene, then each sibling of the affected child has a 25% chance of being affected with SD.^[1]^ Once pathogenic variants of HEXB are identified in the affected family members, prenatal/preimplantation genetic testing can be performed for screening of SD in cases of pregnancies of high-risk relatives.^[2]^ HEXA mutations cause Tay-Sachs disease (TSD). The HEXB gene has been mapped to chromosome 5q13 and contains 14 exons; mutations in HEXB are known to cause SD.^[3,4]^

## 2. Case report

The patient was a 31-month-old Chinese male with a homozygous frameshift variant c.118delG (p.A40fs*24) of the HEXB gene. He was admitted to our hospital in October 2019 because he was unable to walk unsupported. The child was found to have shown exaggerated startle reactions during infancy, movement retrogression at 2 years of age, and reduced limb activity. He underwent rehabilitation treatment for 1 year at a local rehabilitation hospital with no improvement in physical or language skills.

The child was in good health and had no abnormal birth conditions. The child’s mother was G2P1 and had a spontaneous delivery. The child was born without oxygen deprivation or asphyxia, and the umbilical cord was not wrapped around his neck. The birth weight was 3.4 kg, and the birth height was 50 cm. The parents were healthy, and the mother did not have any special medical history during pregnancy. The child received mixed feeding after birth. At 3 months of age, he could raise his head; at 7 months, he could sit independently; at 10 months, he started crawling; at 12 months, he could stand with the support of external objects; at 15 months, he could speak single words; so far, he could not walk unsupported.

A detailed physical examination of the patient was performed. The general state of the child was normal, but he exhibited poor communication skills. Moreover, he had telecanthus. No abnormalities were found in the heart, lungs, abdomen, spinal extremities, and external genitalia. A high degree of muscular tension was observed in the limbs (strength IV). Bilateral knee and Achilles tendon reflexes were hyperactive. Ankle clonus and bilateral Babinski sign were positive. All the meningeal stimulation signs were negative. To observe whether the patient had typical ophthalmologic signs, we suggested a specialist examination, but the family refused our request.

Description of the patient’s gross motor skills: He could stand with the support of external objects but could not stand or walk independently. His head was tilted backward. A high degree of muscular tension of the limbs and tiptoe walking (the right foot was worse) were observed; contracture of the right Achilles tendon was observed. Poor hip and knee flexion and extension were noted.

Description of the patient’s fine motor skills: The patient could grasp, place, and pinch several items, and build 2 to 3 building blocks using both upper limbs.

Description of the patient’s intellectual abilities: He was able to discern when his name was spoken and could respond to the name-calling. He exhibited poor language comprehension skills, his pronunciation lacked clarity, and he spoke single words instead of sentences.

The patient’s routine blood and urine test results, liver and kidney function test results, and myocardial enzyme, lactic acid, and homocysteine levels were normal. The results of urine and blood analyses by gas chromatography coupled with tandem mass spectrometry were normal. Abnormalities were not detected in the electrocardiographic examination. However, magnetic resonance imaging of the head revealed brain atrophy and delayed myelination of the white matter of the brain. The Gesell Developmental Schedules score was 38.96.

**Figure 1.**
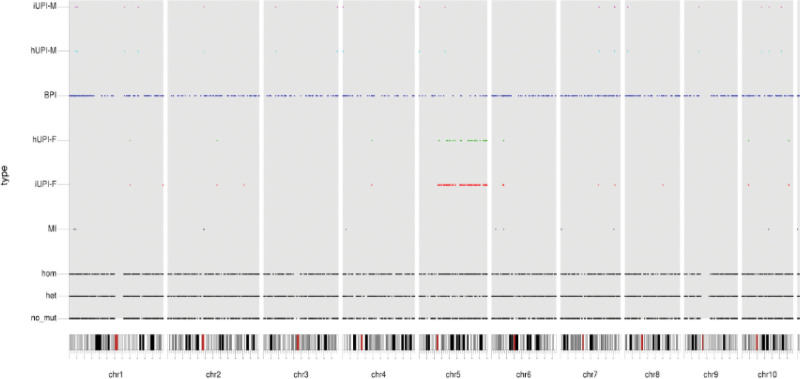
WES was conducted using blood samples. Here, the result of the analysis of the paternal uniparental disomy of the patient is shown. WES = whole-exome sequencing.

To confirm the diagnosis, we performed whole-exome sequencing (WES) using blood samples collected from the proband and his parents. The analysis revealed a novel frameshift mutation, c.118delG (p.A40fs*24), in the HEXB gene. The child’s father carried the heterozygous variants in the same location, but not his mother (Fig. 1).

To guarantee the patient’s rights, we obtained written informed consent from the patient’s guardian. DNA was extracted from peripheral blood samples collected from the patient and his parents. This patient’s genetic model was considered as paternal uniparental disomy. The standards and guidelines prescribed by the American College of Medical Genetics and Genomics were followed for the interpretation of sequence variation and comprehensive evaluation of the pathogenicity of the mutated sites.^[5]^ Analysis of the general population database revealed that it was a low-frequency variant; it was a null mutation, which might have led to the loss of gene function observed in the patient.

In February 2020, at the age of 3 years, the child had a seizure and was treated with oral levetiracetam. At present, the child is 5 years old, and his motor abilities are significantly regressed; thus, he is unable to sit or stand independently.

## 3. Discussion

In this case, a frameshift homozygous variant of the HEXB gene, c.118delG (p.A40fs*24), resulted in deficiencies in motor abilities and language skills and orbital hypertelorism. This variant has not been previously reported in the literature. The child was diagnosed with SD.

The GM2 ganglionopathy includes 3 diseases, such as the TSD, SD, and AB variants (Online Mendelian Inheritance in Man: 272750). The TSD and SD are caused by mutations in the genes of the α- and β-subunits encoding the Hex enzyme, HEXA, and HEXB enzymes. A deficiency of Hex enzymes causes the accumulation of GM2 gangliosides in various cells and tissues of the body, mainly in the central nervous system. The 3 disorders are clinically similar and are characterized by cognitive and motor dysfunction.

SD is a progressive neurodegenerative disease caused by mutations in the HEXB gene present on chromosome 5q13 and was first described by Sandhoff et al.^[6]^ It is a severe lysosomal storage disorder, accounting for 7% of the cases of GM2 ganglioside disease, in which deficiency of hexosaminidase A or B enzymes leads to the accumulation of glycosphingolipids in neuronal cells.^[7]^ It is an autosomal recessive genetic disease, and therefore, males and females are equally affected by this disease.^[8]^ According to the age of onset of symptoms, the disease is categorized into 3 types: classic infantile, juvenile, and adult late onset. The classic infantile form is the most common form of this disease. Around 180 types of HEXB gene mutations have been reported so far, including missense/nonsense and splicing mutations, small deletions, and gross deletions; among these, missense and nonsense mutations are the most commonly reported types.^[9]^ A frequently observed mutation is c.445 + 1G>A, which occurs at a conserved intron site and leads to the complete loss of a canonical donor splice site.^[10]^

The clinical manifestations of infantile SD are progressive neurological impairment, hyperacusis, generalized hypotonia, bilateral cherry-red spots in the eyes, and tonic-clonic or myoclonic seizures. Symptoms generally appear between 3 and 9 months, such as inability to hold the head up, inability to sit unsupported, reduced limb movements, hypotonia, paralysis, blindness, and difficulty in swallowing. In the present case, regression of the child’s motor skills was noticed at age 2 years, and generalized hypotonia and epilepsy were observed at age 3 years; these symptoms were different from the typical symptoms observed in cases of infantile SD, and more in line with Subacute juvenile Sandhoff disease: most of the age of onset was 2 to 5, after reaching normal developmental milestones, developmental progression slows down, followed by developmental degeneration and neurological dysfunction, like gait abnormalities, dysarthria, and cognitive decline.

As the patient exhibited regression in motor skills, a basic laboratory examination was conducted. We suspected that the child had a genetic disease; hence, WES was performed. Analysis of the WES results revealed homozygous frameshift mutations in the HEXB gene, which were predicted to be pathogenic according to the American College of Medical Genetics and Genomics guidelines. Mutations in the HEXB gene can cause SD, and the genotype of our patient corresponded with the clinical phenotype of SD. We presented further Hexosaminidase enzyme testing to make a clear diagnosis, but unfortunately, the patient’s guardian declined our request.

The heterozygous variant c.118delG(p.A40fs*24) was found in the patient’s father, but the child’s mutation type was homozygous. We consider it might be a paternal uniparental disomy, but also could not rule out whether the patient has other HEXB gene mutation in the same position (although low odds). We will actively communicate with the family, and perfect the relevant inspection for further clarification.

Unfortunately, no specific treatment for SD is currently available, and patients with infantile types of SD usually die before they are 3 years old.^[11]^ It has been reported that the administration of miglustat along with a ketogenic diet has a therapeutic effect on adolescents with SD.^[12]^

In conclusion, we have reported a case of SD caused by a homozygous frameshift mutation in the HEXB gene, c.118delG (p.A40fs*24), which has not been previously reported in the literature. The main clinical manifestations in the child were regression in motor abilities beginning at 2 years of age, orbital hypertelorism, and seizures at age 3 years. Magnetic resonance imaging of the head showed cerebral atrophy and delayed myelination of the cerebral white matter. In this study, we could correlate the clinical phenotype with the genotype of SD. As the HEXB pathogenic variant has been identified in the affected family member, prenatal or preimplantation genetic testing can be performed to screen high-risk relatives in the future.

## 4. Patient perspective

Although our child couldn’t be the same as other healthy peers after treatment, we have a more detailed understanding of the child’s situation with the help of doctors and have a general psychological expectation of the child’s future development. We believe that with the help of doctors, our child will have a better quality of life.

## Acknowledgments

We would like to thank the patient and her family members for their contribution to this study.

## Author contributions

**Data curation:** Wanqi Wang.

**Project administration:** Yangfan Qi, Xiaochun Feng.

**Validation:** Jiayi Li.

**Visualization:** Yang Chen, Qiandui Chen.

**Writing – original draft:** Hongyan Xie.

**Writing – review & editing:** Shuangzhu Lin.
